# Supplementary material for: Pyruvate kinase variant of fission yeast tunes carbon metabolism, cell regulation, growth and stress resistance
Source: Mol Syst Biol. 2020 Apr 22;16(4):e9270. doi: 10.15252/msb.20199270 (PMC7175467; doi:10.15252/msb.20199270)
Supplement: Supplementary file 1 — Appendix [file MSB-16-e9270-s001.pdf]

APPENDIX

Pyruvate kinase variant of fission yeast tunes carbon metabolism triggering systemic changes in cell regulation, growth and stress resistance

Stephan Kamrad*[1,2], Jan Grossbach*[3], Maria Rodríguez-López[2], Michael Mülleder[1,6], StJohn Townsend[1,2], Valentina Cappelletti[4], Gorjan Stojanovski[2], Clara Correia-Melo[1], Paola Picotti[4], Andreas Beyer[3,5,#], Markus Ralser[1,6,#], Jürg Bähler[2,#]

[1]The Francis Crick Institute, Molecular Biology of Metabolism Laboratory, London, United Kingdom.
[2]University College London, Institute of Healthy Ageing and Department of Genetics, Evolution & Environment, London WC1E 6BT, United Kingdom.

[3]CECAD, University of Cologne, Cologne, Germany.
[4]Institute of Molecular Systems Biology, Department of Biology, ETH Zurich, Zurich, Switzerland.

[5]Center for Molecular Medicine Cologne, Cologne, Germany.
[6]Charité University Medicine, Berlin, Germany.

*These authors contributed equally

#Corresponding authors

<u>Table of contents</u>:

Appendix Figure S1 Page 2
Appendix Figure S2 Page 3
Appendix Figure S3 Page 4
Appendix Figure S4 Page 5
Appendix Figure S5 Page 6
Appendix Figure S6 Page 7
Appendix Figure S7 Page 8
Appendix Figure S8 Page 9
Appendix Table S1 Page 13
Appendix Table S2 Page 14

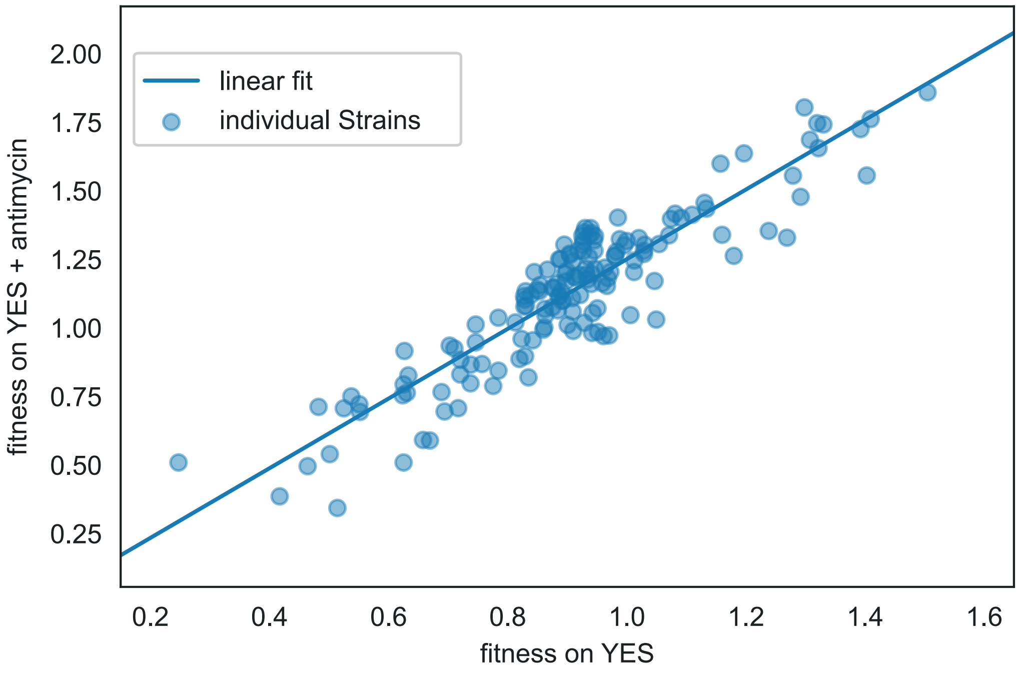

Appendix Figure S1: Fitness (corrected colony size relative to standard lab strain 972) of wild strains on YES and YES with 500ug/L antimycin A. A linear regression with $y = 1.27x - 0.02$ describes this relationship well ($R^2 = 0.83$) which means most of the variation of fitness on YES + antimycin is explained by basal growth rate and not specific to the effect of antimycin. Antimycin resistance is determined by taking the ratio of the fitness of YES + antimycin and YES alone.

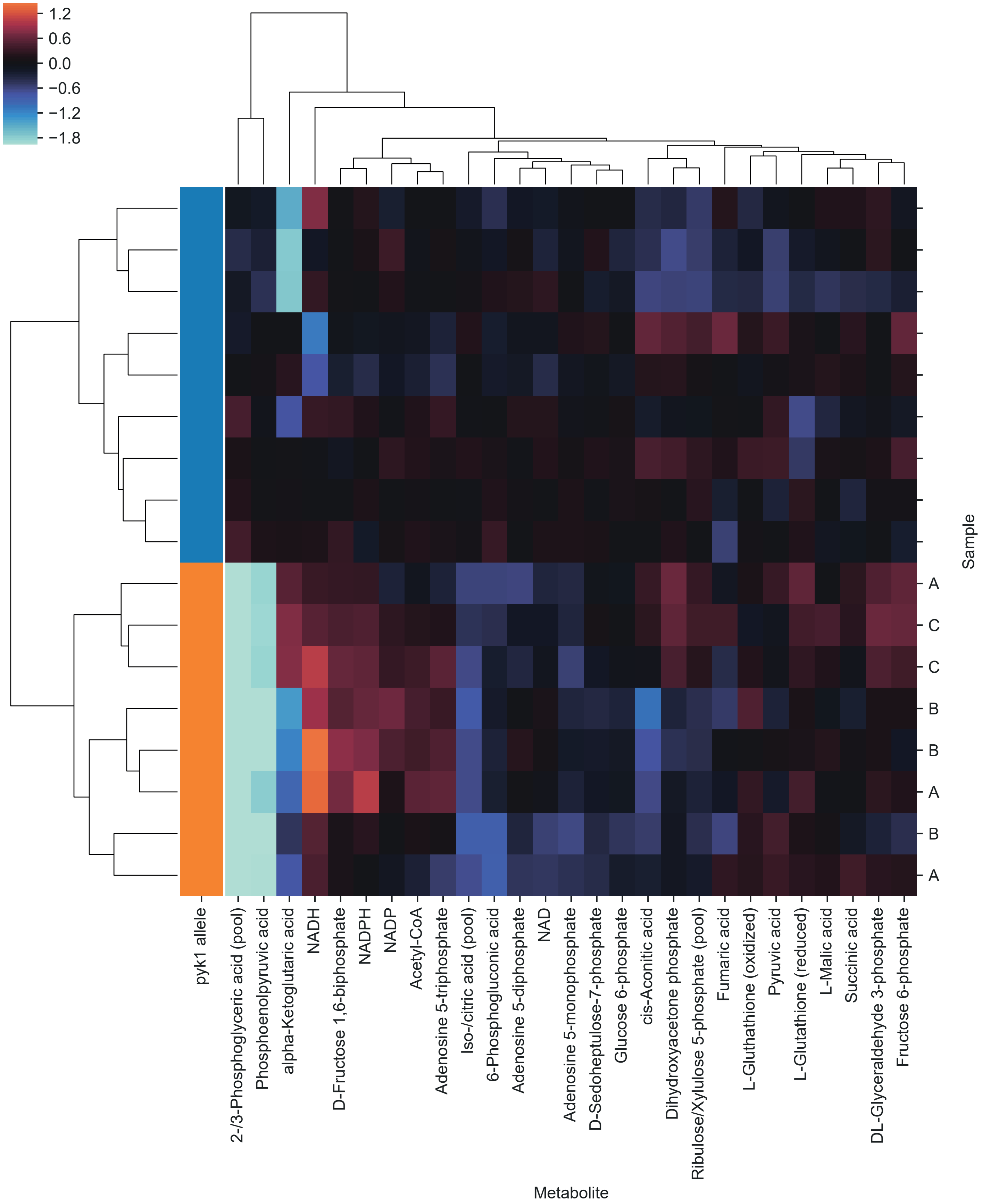

Appendix Figure S2: Clustermap of metabolite data for T and A strain. Data was normalised by dividing by the median of the T-strain and subsequently log2-trabsformed. Clustering was performed based on Euclidean distance with the average method. The colourbar was clipped at -2 in the negative direction to aid visualisation. The pyk1 allele is shown for each row, with organge and blue corresponding to the A- and T-allele respectively. Row labels on the right denote the independent mutant strains.

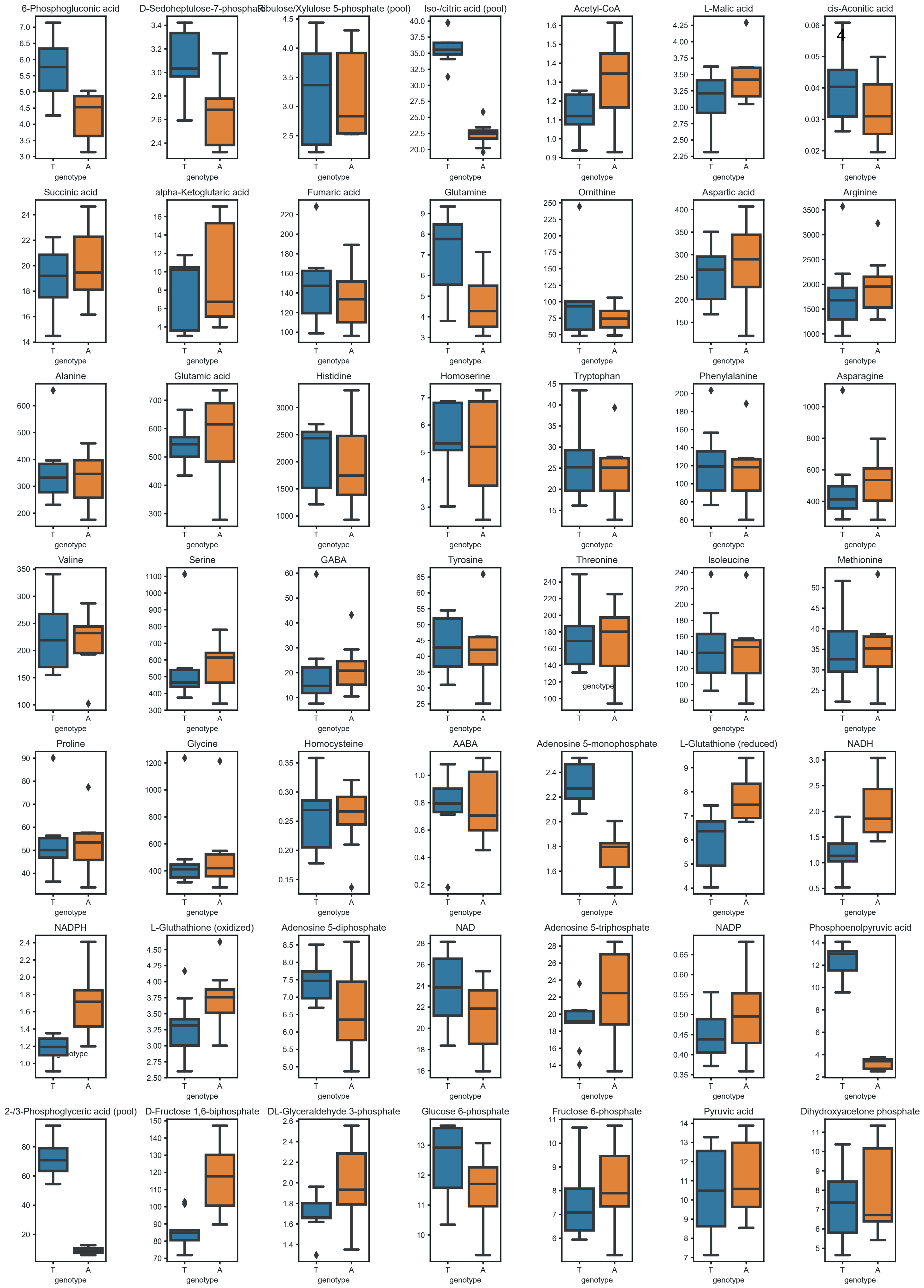

Appendix Figure S3: Boxplots comparing intracellular metabolite concentrations (concentrations in prepared sample divided by optical density of culture at sampling) of T-strain (*S. pombe* reference strain 968) and A-strain (968 pyk1^{T343A}).

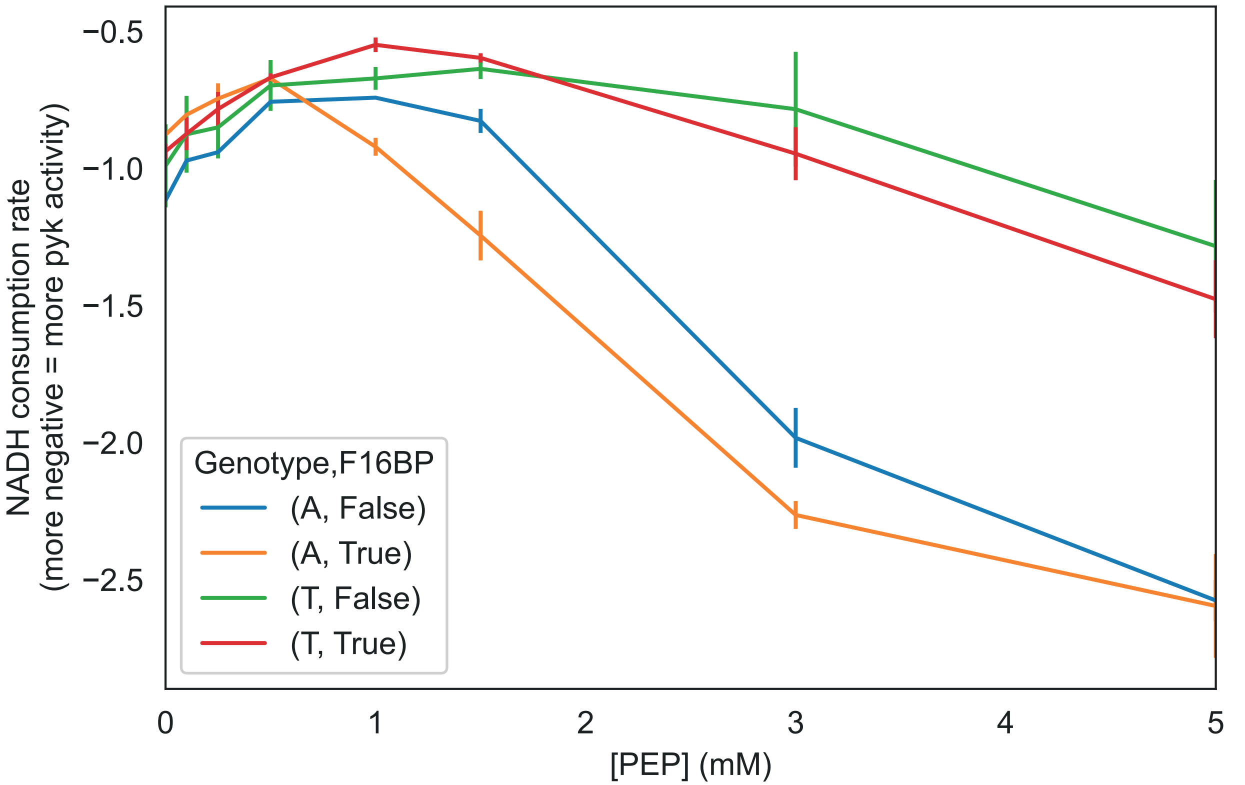

Appendix Figure S4: Substrate conversion rates depending on the initial concentration of phosphoenol pyruvate in the assay mix, with and without 5mM fructose 1,6-bisphosphate. Fresh cell lysates from three biological replicates were used, error bars show standard deviations.

(A)

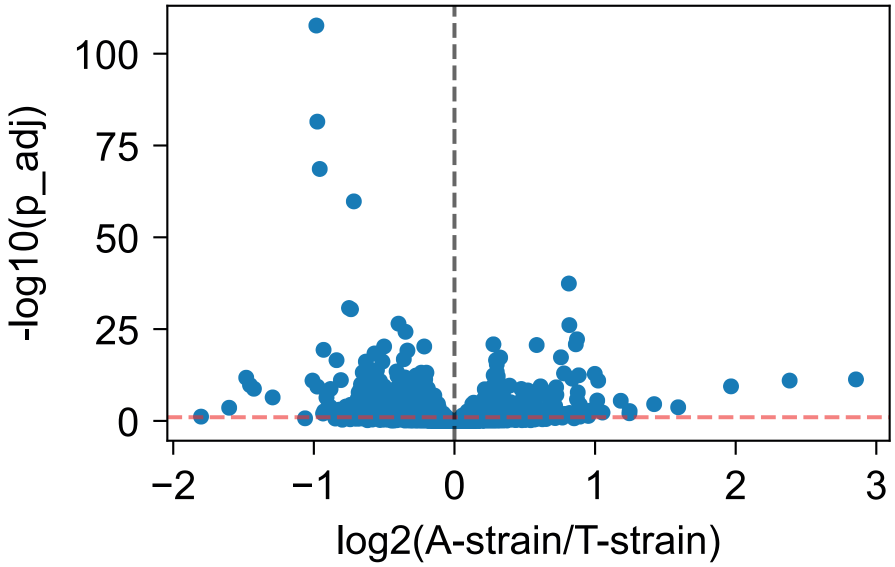

(B)

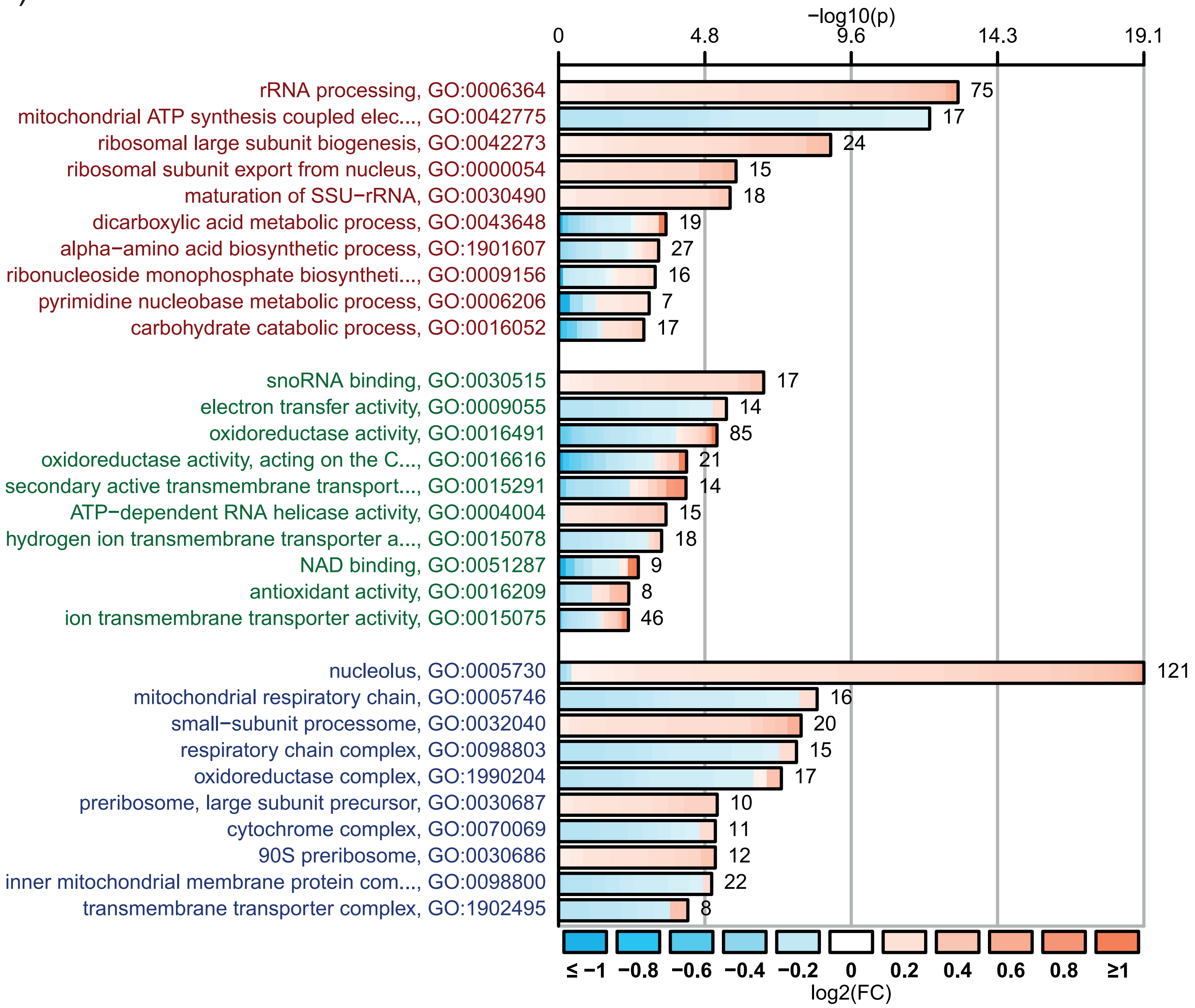

Appendix Figure S5: (A) Volcano plot of transcriptome data set. (B) CellPlots summarising GO enrichment analysis of transcripts differentially expressed in A-strain versus T-strain.

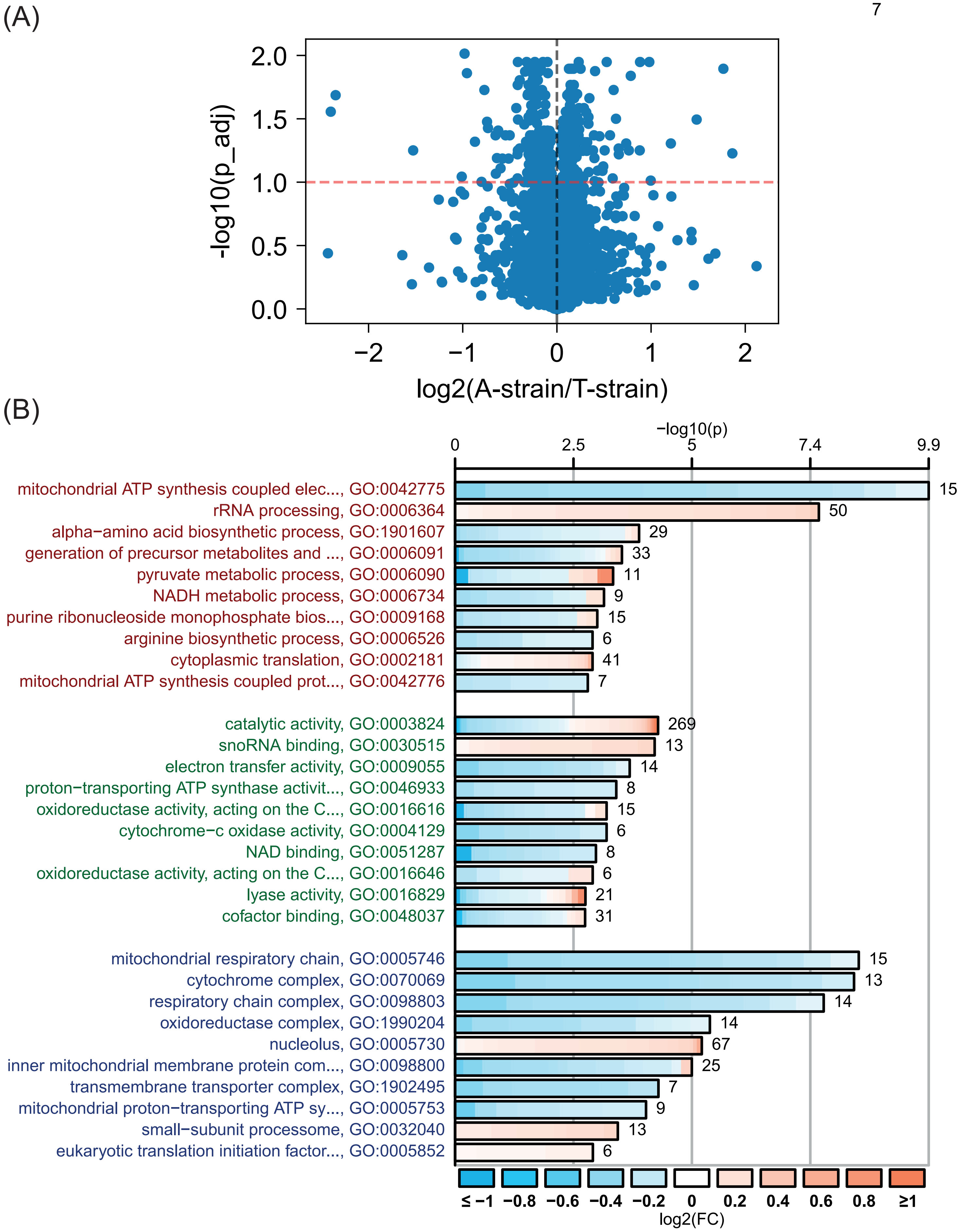

Appendix Figure S6: (A) Volcano plot of proteome data set. (B) CellPlots summarising GO enrichment analysis of proteins differentially expressed in A-strain versus T-strain.

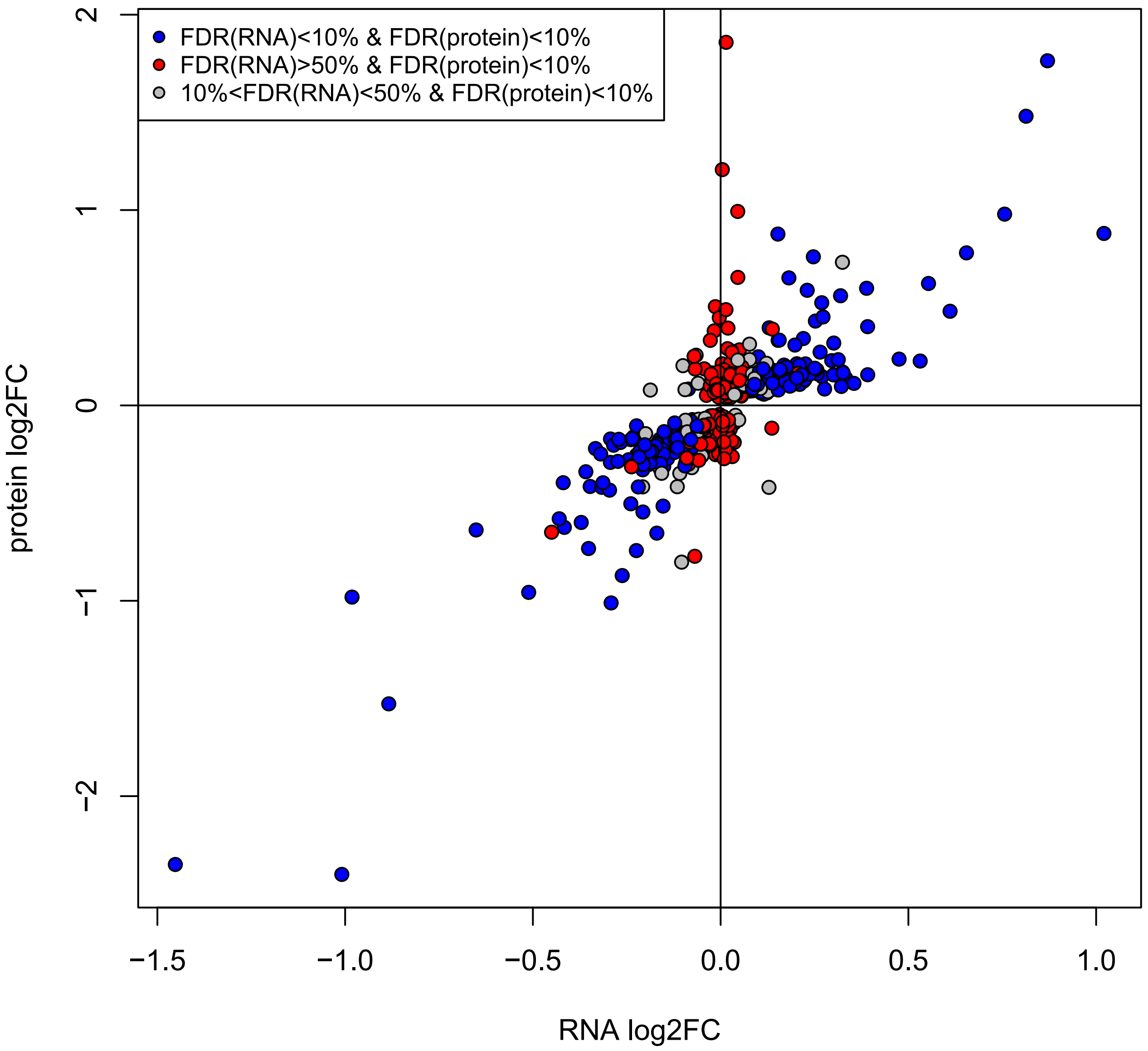

Appendix Figure S7: Differential abundance of transcripts and proteins. All genes with differential protein abundance between the A-strain and the T-strain at FDR<10% are shown. Genes for which the transcripts were not deferentially abundant (FDR>50%) are shown in red. Genes whose transcripts and proteins were deferentially abundant at FDR<10% are shown in blue. Fold-changes were computed as log2(A)-log2(T). Genes that were only affected on the protein level (red) were significantly enriched in functions related to cytoplasmic translation and depleted in genes involved in ribosome biogenesis compared to genes affected on both levels (blue, Appendix Tables S5 and S6).

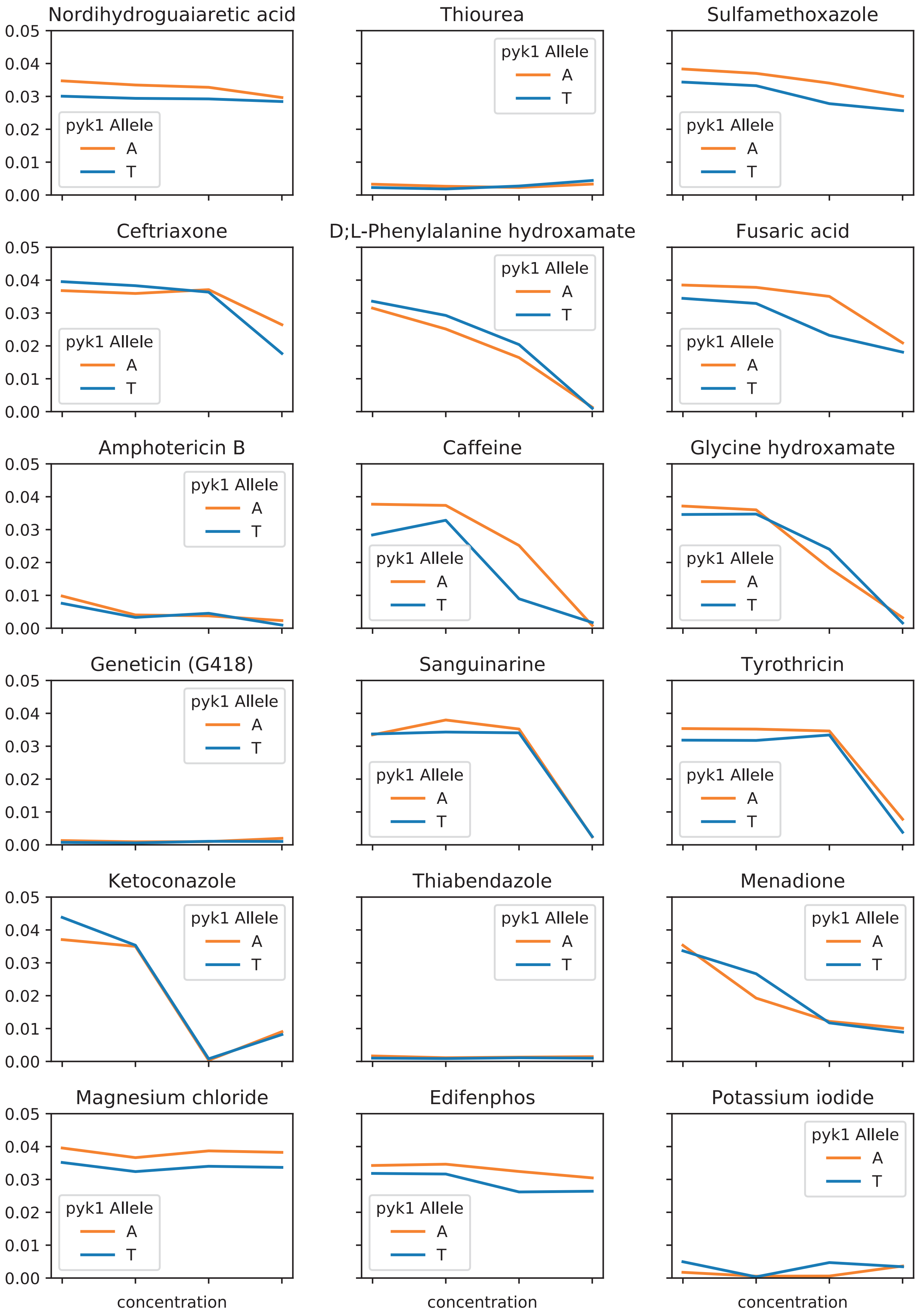

Appendix Figure S8 (Page 1 of 4): Dose-response curves of T- and A-strain, showing maximum growth rate (y-axis) for each of the 4 concentration levels contained in the Biolog Phenotype Array.

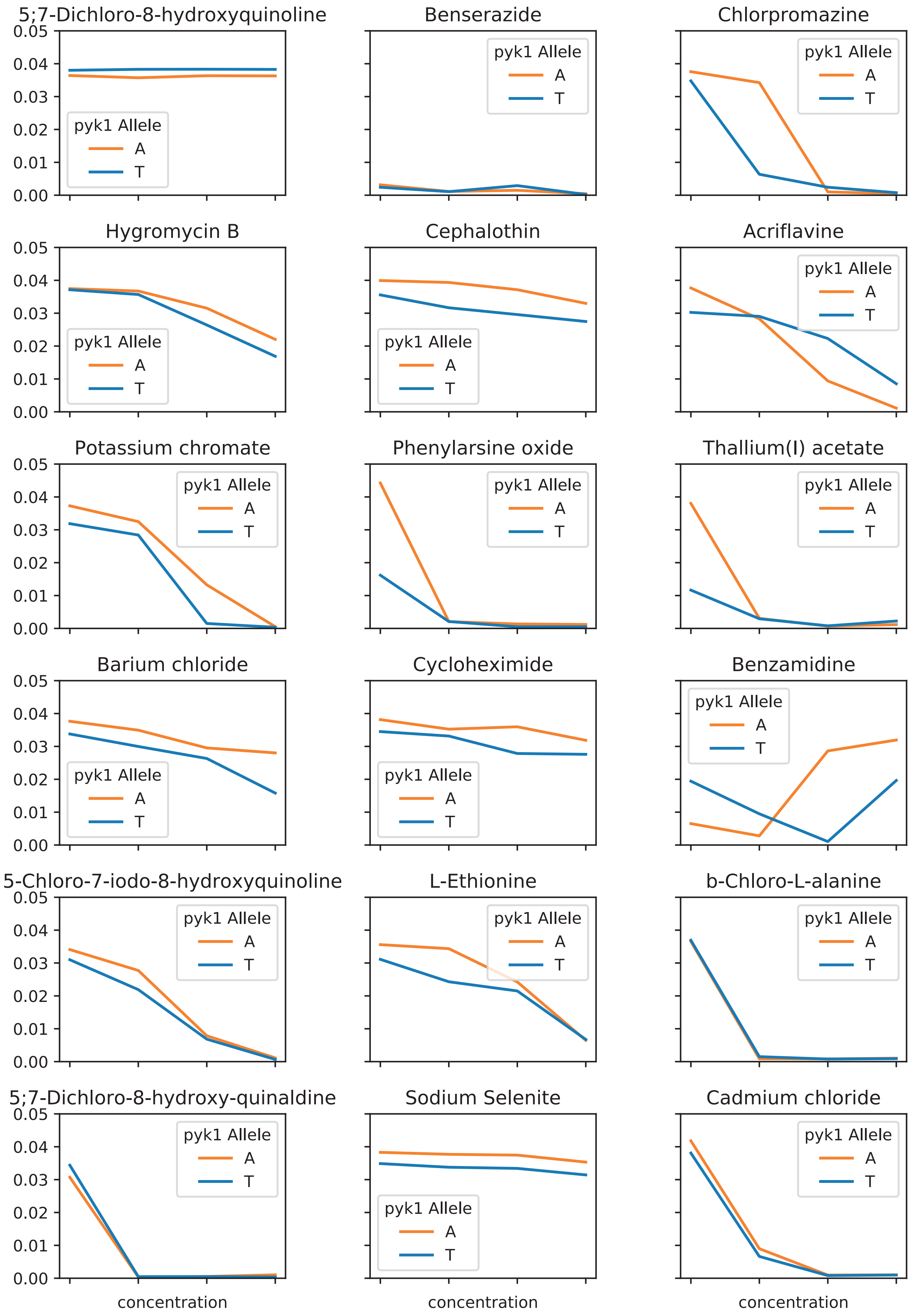

Appendix Figure S8 (Page 2 of 4): Dose-response curves of T- and A-strain, showing maximum growth rate (y-axis) for each of the 4 concentration levels contained in the Biolog Phenotype Array.

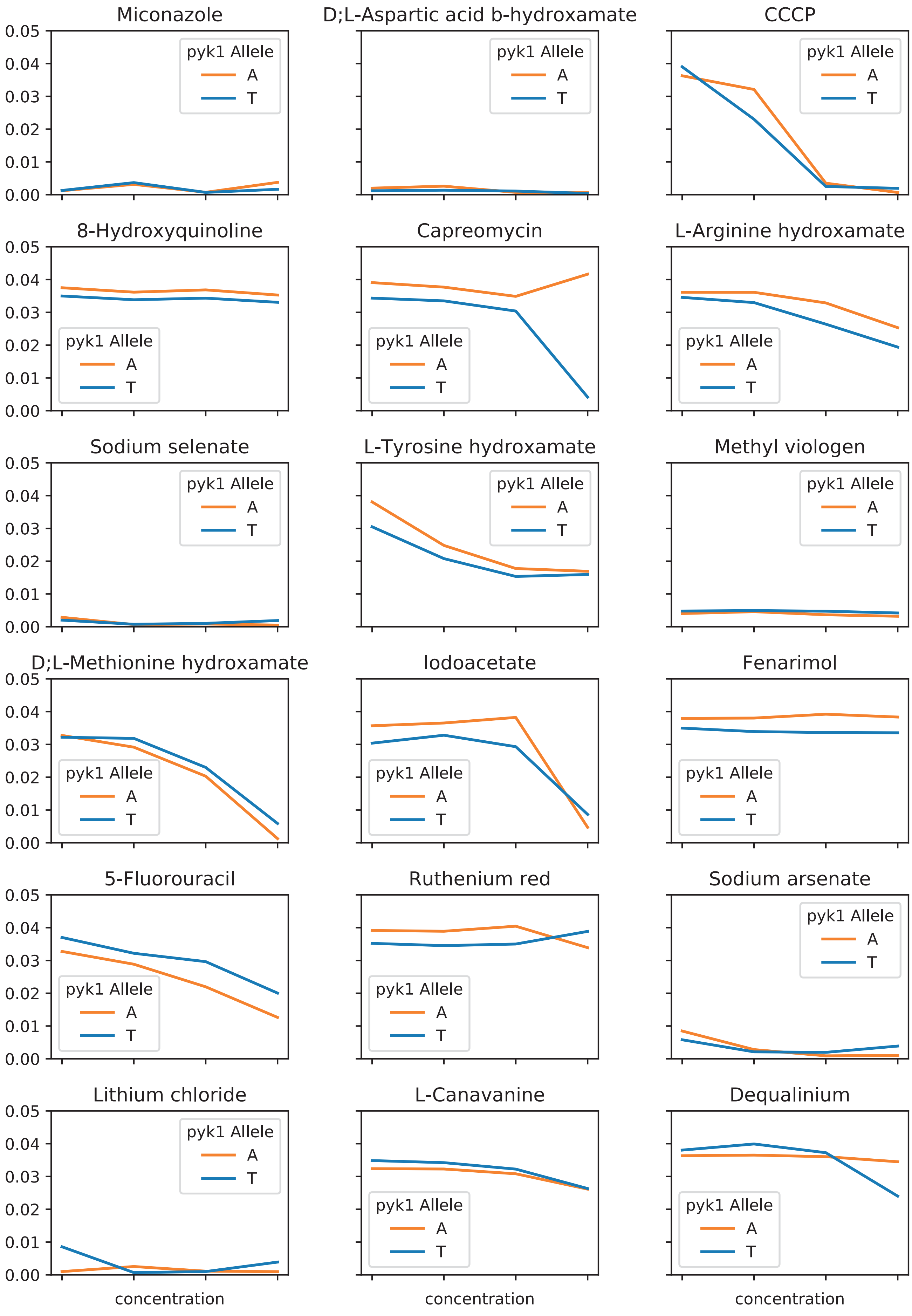

Appendix Figure S8 (Page 3 of 4): Dose-response curves of T- and A-strain, showing maximum growth rate (y-axis) for each of the 4 concentration levels contained in the Biolog Phenotype Array.

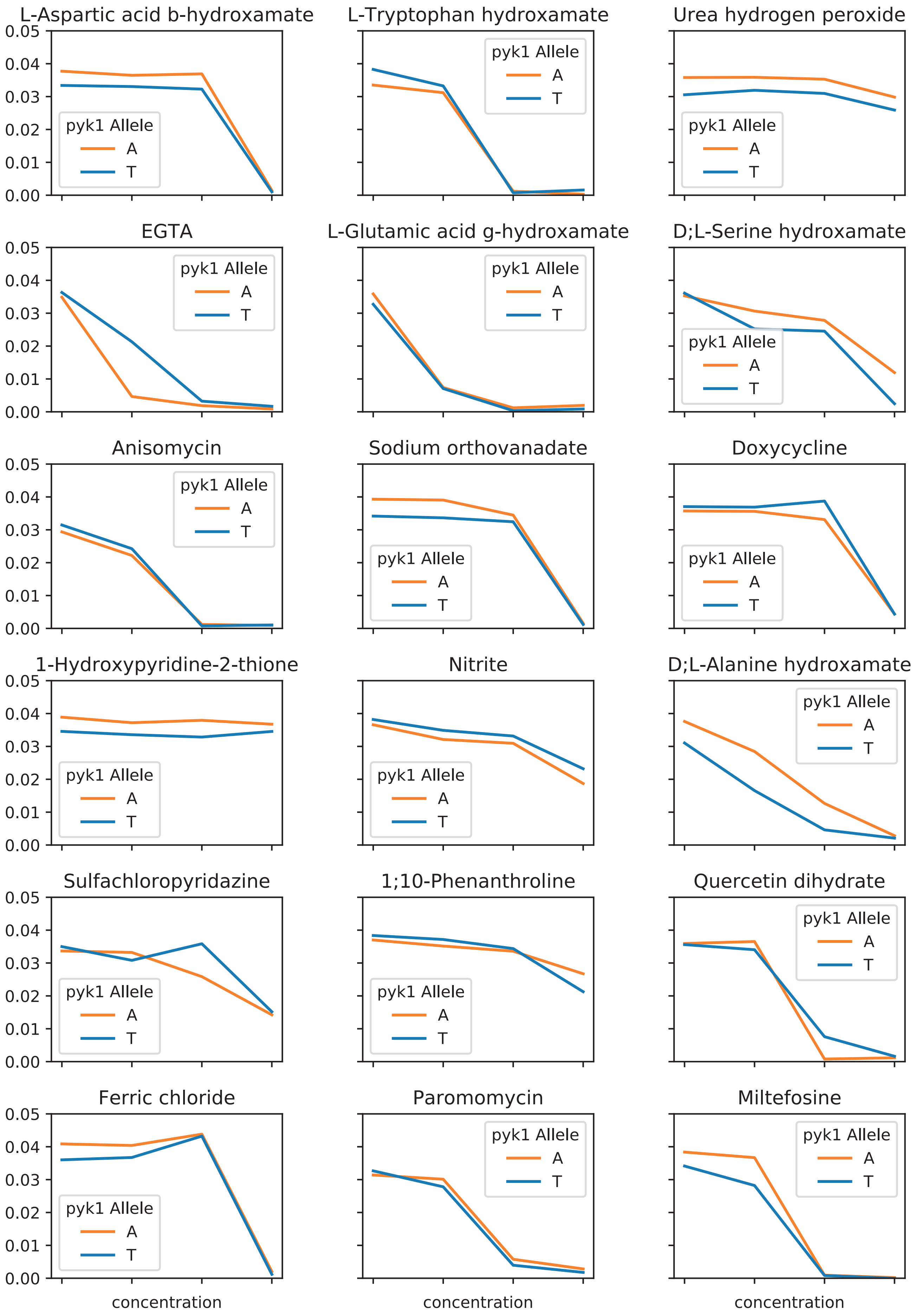

Appendix Figure S8 (Page 4 of 4): Dose-response curves of T- and A-strain, showing maximum growth rate (y-axis) for each of the 4 concentration levels contained in the Biolog Phenotype Array.

Ontology	GO.ID	Term	Annotated	Significant	Expected	p-value
BP	GO:0048522	positive regulation of cellular process	13	13	5.24	5.20E-06
BP	GO:0002181	cytoplasmic translation	31	23	12.49	6.70E-05
BP	GO:0034645	cellular macromolecule biosynthetic proc...	61	44	24.57	0.00012
BP	GO:0044267	cellular protein metabolic process	64	44	25.78	0.00096
BP	GO:0033043	regulation of organelle organization	10	9	4.03	0.00156
BP	GO:0051276	chromosome organization	15	11	6.04	0.00864
MF	GO:0003735	structural constituent of ribosome	14	13	5.64	4.70E-05
MF	GO:0005515	protein binding	42	29	16.92	5.80E-05
CC	GO:0022626	cytosolic ribosome	14	13	5.64	4.70E-05
CC	GO:0015934	large ribosomal subunit	10	9	4.03	0.0016
CC	GO:0044391	ribosomal subunit	15	14	6.04	0.0084
CC	GO:0005737	cytoplasm	297	129	119.65	0.0089

Appendix Table S1: GO-enrichments of genes regulated only on the protein level
GO-enrichments of genes regulated only on the protein level at FDR<10% (FDR for transcripts > 50%, red dots in Appendix Figure S5) compared to genes that were regulated on both the transcript and protein level at FDR<10% (blue dots in Appendix Figure S5).

Ontology	GO.ID	Term	Annotated	Significant	Expected	p-value
BP	GO:0006364	rRNA processing	39	35	23.29	1.50E-05
BP	GO:0046034	ATP metabolic process	26	22	15.53	0.0047
BP	GO:0098662	inorganic cation transmembrane transport	10	10	5.97	0.0053
MF	GO:0015078	hydrogen ion transmembrane transporter a...	17	17	10.15	0.00012
MF	GO:0016491	oxidoreductase activity	54	43	32.25	0.0007
CC	GO:0005730	nucleolus	57	48	34.04	1.60E-05
CC	GO:0030684	preribosome	18	17	10.75	0.00097
CC	GO:0098800	inner mitochondrial membrane protein com...	21	19	12.54	0.00171

Appendix Table S2: GO-enrichments of genes regulated on both the transcript and protein level
GO-enrichments of genes regulated on both the transcript and protein level at FDR<10% (blue dots in Appendix Figure S5) compared to genes that were regulated only on the protein level at FDR<10% (FDR for transcripts > 50%, red dots in Appendix Figure S5).
